# Improving the Mechanical, Thermoelectric Insulations, and Wettability Properties of Acrylic Polymers: Effect of Silica or Cement Nanoparticles Loading and Plasma Treatment

**DOI:** 10.3390/polym16212965

**Published:** 2024-10-23

**Authors:** Seenaa I. Hussein, Saba J. Kadhem, Nadai A. Ali, Alhafez M. Alraih, Alaa M. Abd-Elnaiem

**Affiliations:** 1 Department of Physics, College of Science, University of Baghdad, Baghdad 10071, Iraq; seenaa.hussein@sc.uobaghdad.edu.iq (S.I.H.); dr.sabakadhem@gmail.com (S.J.K.); nadia.ali@sc.uobaghdad.edu.iq (N.A.A.); 2 Department of Chemistry, College of Science and Arts, Mohail Aseer, King Khalid University, Abha 62529, Saudi Arabia; alalshafee@kku.edu.sa; 3 Physics Department, Faculty of Science, Assiut University, Assiut 71516, Egypt

**Keywords:** acrylic polymer composites, plasma jet, cement, silica, hydrophobic, mechanical, thermoelectric

## Abstract

The acrylic polymer composites in this study are made up of various weight ratios of cement or silica nanoparticles (1, 3, 5, and 10 wt%) using the casting method. The effects of doping ratio/type on mechanical, dielectric, thermal, and hydrophobic properties were investigated. Acrylic polymer composites containing 5 wt% cement or silica nanoparticles had the lowest abrasion wear rates and the highest shore-D hardness and impact strength. The increase in the inclusion of cement or silica nanoparticles enhanced surface roughness, water contact angle (WCA), and thermal insulation. Acrylic/cement composites demonstrated higher mechanical, electrical, and thermal insulation properties than acrylic/silica composites because of their lower particle size and their low thermal/electrical conductivity. Furthermore, to improve the surface hydrophobic characteristics of acrylic composites, the surface was treated with a dielectric barrier discharge (DBD) plasma jet. The DBD plasma jet treatment significantly enhanced the hydrophobicity of acrylic polymer composites. For example, the WCA of acrylic composites containing 5 wt% silica or cement nanoparticles increased from 35.3° to 55° and 44.7° to 73°, respectively, by plasma treatment performed at an Ar flow rate of 5 L/min and for an exposure interval of 25 s. The DBD plasma jet treatment is an excellent and inexpensive technique for improving the hydrophobic properties of acrylic polymer composites. These findings offer important perspectives on the development of materials coating for technical applications.

## 1. Introduction

Coating systems should be compatible with the surface since incompatible coatings might result in failures during application even for short lifetimes [1,2,3]. The failures may occur immediately after application because of the incompatibility of solvents or due to wetting issues [4]. The coating properties may include resistance to exterior weathering such as water chemical resistance, thermal resistance, abrasion, etc. [4,5]. Polymer-modified cement, which serves as a hydraulic binder, has long maintained human civilizations [6]. Geopolymers can be improved as an alternative to regular Portland cement by a range of coatings such as higher compressive strength, hydrophobic, and antibacterial coatings [7,8]. Nevertheless, because these coatings are employed on a large scale, their surfaces should be hydrophilic, or wettable, for the coating to spread and adhere properly [9]. The surface wettability of coating polymer materials such as acrylic ey polymers can be controlled by their composition, polymerization duration, surface energy, and secondary treatments [10,11,12]. Usually, the polymers are incorporated with numerous nanofillers, including graphene, carbon nanotubes (CNTs), ZrO_2_, TiO_2_, SiO_2_, etc., to form polymer nanocomposites [13,14,15,16,17,18,19]. The incorporation of nanofiller usually affects the mechanical, electric, chemical, and thermal characteristics due to the interlocking/adhesion between the nanofiller and the polymer matrix [3,16].

Flexible and stiff waterproof materials are common types of waterproofing materials [20,21]. Traditional flexible waterproof materials, such as coil and coating, have been shown to be flexible and durable in practice, but they are weak at adhesion, unsuitable for wet base surfaces, and not suited for surface and wet surface applications [22,23]. On the other hand, rigid waterproofing materials containing cement are usually desirable due to their greater performance compared with flexible waterproofing materials [24]. Nevertheless, the majority of stiff waterproof compounds are surface-sealing waterproofing agents, with the influence of waterproofing acting only on the surface [3,9,18]. It has little effect on waterproofing over time since it cannot automatically and completely penetrate the internal structure [25]. Besides the mechanical properties, the wettability properties of acrylic polymers were enhanced by adjusting the concentration of doped fillers such as cement and silica, among other fillers [4,18]. The surface of a polymer can typically be manipulated and modified by incorporating various nanomaterials into the main polymer matrix, but certain difficulties are expected to arise during this process [26,27]. The physical characteristics of polymer-based composites may be enhanced using different coating materials and manufacturing processes [28]. Coatings are frequently employed across many sectors, and there is a need to develop a reliable technique for assessing the mechanical characteristics of coatings and adhesives [29]. Different strategies have been explored to enhance the mechanical characteristics of adhesives and coatings [30].

In general, composites are made up of two or more components. The physicochemical properties, specifically the mechanical and thermal characteristics of the composites, are substantially different compared to their individual components [27,31]. These compounds have extraordinary properties such as chemical, thermal, and ultraviolet resistance, as well as excellent adhesion to the majority of surfaces and can, therefore, be used to make pottery (polymer-derived ceramics) [32].

The hydrophobic nature of polymer composites has been modified as a result of several strategies, including plasma treatment, which affects the surface roughness of the materials [33]. Moreover, polymer deposition and surface modification using plasma jets are beneficial for diagnostic equipment and biosensors [34]. Previous research has demonstrated the utilization of plasma to enhance both the electrical conductivity and hydrophobic characteristics of cotton fiber/polypyrrole/Ag nanocomposites [35]. The mechanical properties of cotton fabric are affected slightly by plasma treatment [36,37]. The observed increase in mechanical characteristics after plasma treatment appears to be due to better adhesive bonding quality and adhesion properties [9]. As a result, the use of plasma is advised for surface, structure, and optoelectronic modifications but not for mechanical improvement of polymers [37,38,39,40]. It is well known that plasma, the fourth state of matter, can be obtained by an ionized gas that achieves certain conditions, including quasi-neutrality and collective behavior [41,42]. Surface modification by a dielectric barrier discharge (DBD) plasma jet is fast, simple, and environmentally safe. It is also a high-efficiency technique; therefore, this method was adopted in the present work [10]. The application of DBD plasma in treating the surface of acrylic composite polymers serves a strategic purpose in modifying surface properties [35]. Plasma treatment enhances the surface chemistry of acrylic coatings and improves adhesion to the substrate [37]. In addition, the plasma treatment can strengthen the bond between the filler and polymer matrix, which is essential for long-term durability. In general, covalent bonds may form as a result of plasma treatment, which modifies the surface chemistry of the polymer and allows for strong chemical bonding between the polymer chains and functional groups on the nanoparticles [2]. Additionally, van der Waals forces contribute to the adhesion, facilitating more cohesive interlocking of the nanoparticles within the polymer matrix [15]. Moreover, the introduction of hydrophobic functional groups during plasma treatment is advantageous in dealing with silica or cement, facilitating water repellency and preventing moisture infiltration [36]. Plasma treatment also induces crosslinking and polymerization reactions, resulting in a more stable surface that is less prone to environmental degradation. Furthermore, plasma treatment of material surfaces assists in the formation of a rougher surface and enhances their hydrophobicity [43,44]. Plasma treatment is usually applied without damaging the surface of the sample or leaving toxic residues on it [10].

The objective of this work was to improve the mechanical performance and thermal/electrical insulations of acrylic polymers by doping them with cement and silica nanoparticles. The cement filler was selected for this investigation because of its excellent mechanical properties and beneficial wettability with surfaces, as well as its high thermal stability [18]. On the other hand, silica nanoparticles were used as a filler due to their excellent thermal stability, low cost, and availability [16]. The mechanical characteristics (wear rate, impact strength, and Shore-D hardness), thermal insulation, and dielectric strength of acrylic polymers were modified by incorporating cement or silica nanoparticles at various ratios ranging from 0 to 10 wt%. The effect of incorporation ratio and type on the surface roughness and hydrophobic properties of acrylic polymers were investigated. Furthermore, the surface of polymer composites was treated with a plasma jet to modify their hydrophobic properties. The impact of the plasma exposure period and Ar flow rate on surface wettability was studied. The findings are correlated, interpreted, and compared to those in the literature.

## 2. Materials and Methods

### 2.1. Materials

**Figure 1:**
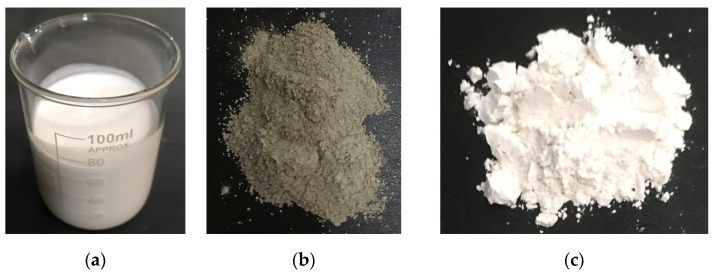
Optical image of (**a**) acrylic polymer solution, (**b**) ordinary Portland cement (OPC) nanopowder, and (**c**) silica (SiO_2_) nanopowder used in this study.

Acrylic polymer solution (Code: D007) and ordinary Portland cement (OPC) nanopowder were acquired from Al Gurg Fosroc LLC Co., Dubai, United Arab Emirates. The acrylic polymer is a milky white polymer with a solid percentage of ~30% at 105 °C and a density of ~1700 kg/m^3^. The OPC is composed of 63 wt% CaO, 21.9 wt% SiO_2_, 6.9 wt% Al_2_O_3_, 3.9 wt% Fe_2_O_3_, 2.5 wt% MgO, and 1.7 wt% SO_3_. Silica (SiO_2_, 99.5%) nanopowder was purchased from Nanjing High Technology Nano Material Co. Ltd., (HTNano), Nanjing, China. Figure 1 illustrates the optical images of these raw materials.

### 2.2. Preparation of Acrylic Polymer Composites

**Figure 2:**
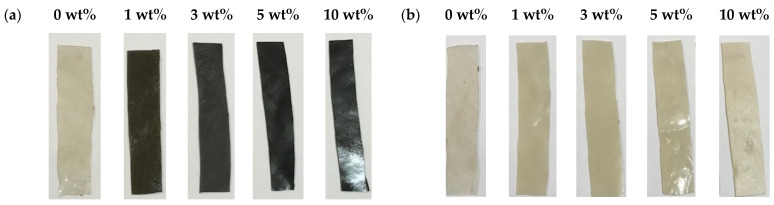
Optical images of (**a**) acrylic/cement and (**b**) acrylic/silica composites.

The acrylic and acrylic composites were synthesized by the casting technique performed at room temperature (RT, ~25 °C). Detailed information about the preparation of acrylic composites by the casting method was described elsewhere [18]. Appropriate weights of cement, or silica nanopowder of 1, 3, 5, and 10 wt% were added to ~20 g acrylic polymer to form polymer composites. The polymer and filler were mixed using a magnetic stirrer at RT for 1 h. Then, the filler and acrylic were further mixed using an ultrasonic shear mixer with a center frequency of 10 MHz. The produced acrylic composites were then dried at RT for 24 h. The optical pictures of the synthesized acrylic/cement (black samples) and acrylic/silica (beige samples) composites are shown in Figure 2a,b.

The thickness of each synthesized acrylic polymer and acrylic polymer composite was 0.1 cm. The samples were cut into square, circular, and rectangular forms based on the desired measurement. The square edge of the square samples of the acrylic polymer composite was 2 cm for hardness and surface roughness tests and 1 cm for WCA measurements. The acrylic polymer composite’s rectangular samples measured 2 cm × 1 cm for abrasion wear rate and 5.5 cm × 1 cm for impact strength testing. The radius of the acrylic polymer composite samples was 2 cm for dielectric strength and thermal insulation measurements.

### 2.3. Plasma Treatment of the Surface of Acrylic Composites

A plasma jet was used to treat the surface of acrylic polymer composites containing 5 wt% silica or cement nanoparticles. The plasma jet was generated in an Ar atmosphere using a piezoelectric direct discharge device model Piezobrush PZ2 (Regensburg, Germany) at atmospheric pressure, temperature of 34 °C, output power of 10 W, and operation frequency of 28 kHz (RF equivalent). The plasma jet device was outfitted with a near-field nozzle designed for conductive surfaces since the normal nozzle generated visible discharges onto the surface of the polymer composites. The plasma was generated by a system consisting of a T-shaped quartz tube with two entrances: the first entrance was for the high-voltage electrode and the second entrance was for the Ar gas. There was also an outlet for the generated plasma torch. In this system, the flow rate of the pure Ar gas is controlled by a flow meter. The plasma was ignited by an AC source at a high voltage of 7.5 kV. The distance between the polymer surface and the plasma torch was 2.5 cm. Optical emission spectroscopy (OES) was used to diagnose the Ar plasma jet by electronically monitoring the excited gases and the intensities of discharges produced by the DBD plasma jet. The Surwit device, model S3000-UV-NIR, Shanghai, China, was used to record the optical emissions spectra within a wavelength range of 250–950 nm. The optical fiber was kept 1 cm away from the plasma torch and was inclined at an angle of 45°. The spectrum was taken for the plasma generated at Ar flow rates of 1, 2, 3, 4, and 5 L/min.

For this purpose, 20 specimens of acrylic/silica and acrylic/cement composites were treated by scanning them with a plasma torch in both X and Y directions. Also, 5 samples of acrylic/silica and 5 samples of acrylic/cement composites were treated by exposing them to plasma with a variable Ar flow rate for a fixed exposure time of 25 s. As for the others, they were treated by exposing them to plasma with exposure durations of 5, 10, 15, 20, and 25 s at a fixed Ar flow rate of 5 L/min.

### 2.4. Characterizations of Acrylic Polymer Composites

#### 2.4.1. Surface Morphology

An atomic force microscope (AFM), model AA3000, manufactured by Angstrom Advanced Inc. in the, Stoughton, MA, USA, was utilized to investigate the surface morphology, particle size, and distribution of loaded silica and cement nanopowder. The surface morphology of acrylic composites was evaluated using a scanning electron microscope (SEM), model Inspect S50 (FEI_Company/Eindhoven, The Netherlands), operated at 30 kV.

#### 2.4.2. Abrasion Wear Resistance

The abrasion wear rate test was performed on pin-on discs with a rotation of 500 rpm at 25 °C. The weight of the tested acrylic composites was measured before (*W*_1_) and after (*W*_2_) the test, and the weight difference (loss), ∆*W*, was utilized to estimate the wear rate. The abrasion wear rate was measured at loads of 5, 10, and 15 N, while the sliding time was fixed at 60 s. The value of the wear rate (g/cm) for the investigated acrylic composites was calculated using the following formula: $wear\;rate=\frac{\Delta W}{2\pi rnt}$, where *r* is the specimen (disc) radius (cm), *n* is the number of runs of the disk (r/min), and *t* is the testing period (min). The wear rate test was conducted three times under the same conditions and the average result was taken.

#### 2.4.3. Impact Strength Test

The impact test is applied to check the ability to absorb energy during the collision of the tested samples. The value of the absorbed energy can be used to estimate various mechanical parameters such as impact strength, toughness, fracture resistance, impact resistance, and fracture resistance of the investigated compounds [4]. In the present study, the impact strength was estimated using the Charpy impact tester, model Charpy–Izod, from Testing Machine Inc., Amityville, NY, USA. The impact strength was measured using the ISO-179 standard [45] test technique. The Charpy impact test (unnotched sample impact test) has a constant speed of 3.4 m/s. Impact strength ($G_{c}$) was calculated for various composites from the following relationship: $G_{c}=\frac{U_{c}}{A}$, where $U_{c}$ is the impact energy (J) and *A* is the cross-section area of the tested material (m^2^).

#### 2.4.4. Shore-D Hardness

The hardness of a material is defined as its resistance to prolonged indentation. There are numerous types of hardness tests that can be applied depending on the size of the materials. In this study, the Shore-D hardness test was applied. The indenter was quickly pressed down using an ASTM D2240 [46] hardness tester, and the highest reading on a digital scale was recorded.

#### 2.4.5. Dielectric Strength

The dielectric strength was used to assess the electrical insulation’s durability. The dielectric strength was measured with an AC (50 Hz) power supply at high voltages (0–60 kV) using BAUR-PGO-S3, manufactured in Hanau, Germany. The device includes oil with a high dielectric strength of 40 kV/mm to prevent the circumstantial spark from being transmitted. In the setup circuit, the poles are made up of spherical Cu plates with a diameter of 0.2 cm. The polymer composite was immersed in oil between the poles to ensure that the poles were in contact with the sample surface. Then, voltage was applied until the breakdown voltage was reached. The location where the breakdown occurs may be determined based on the damage caused by the breakdown [47].

#### 2.4.6. Thermal Insulation

Thermal insulation was evaluated by determining the thermal conductivity coefficient of the various samples investigated. Thermal conductivity was determined by Lee’s disk manufactured by Griffin and George, London, UK. The steps applied for measuring thermal conductivity as well as the thermal insulation were described in detail elsewhere [15,48]. The following formulas were used to compute the thermal conductivity coefficient (*K*) using Lee’s disc technique [15]: $K\frac{T_{B}-T_{A}}{d_{s}}=e\left[T_{A}+\frac{2}{r}\left(d_{A}+\frac{d_{S}}{4}\right)T_{A}+\frac{1}{2r}d_{S}T_{B}\right]$, and $H=IV=\pi r^{2}e\left(T_{A}+T_{B}\right)+2\pi re\left[d_{A}T_{A}+\frac{d_{S}\left(T_{A}+T_{B}\right)}{2}+d_{B}T_{B}+d_{C}T_{C}\right]$, where *V* is the applied voltage, *I* is the recorded current, *d* is the disc thickness, *r* is the disc radius, $d_{S}$ is the sample thickness, $d_{A}$, $d_{B}$, and $d_{C}$ are the disk’s respective thicknesses, *T* is the disk’s temperature, and e is the thermal energy transmitted through the disc material per unit area per second.

#### 2.4.7. Surface Roughness

A surface’s roughness is a numerical number that describes the texture of the surface. The roughness of surfaces in this study was investigated by the TR100 surface roughness tester. A general definition of the height variant on surfaces can be derived from the average arithmetic height (*R_a_*) parameter.

#### 2.4.8. Water Contact Angle

The water contact angle (WCA) is the angle formed when a drop of water merges with a solid surface. The WCA is used to quantify the wettability of substrate surfaces. The sessile drop technique is the most often used method for determining the WCA. A syringe pump was used to generate a 5 µm droplet of water at 25 °C on the sample’s surface and the view was captured using a SONY CORP DSC-W310 digital camera, Tokyo, Japan, with a resolution of 12.1 MP. The WCA on the sample surface was estimated using ImageJ software version 1.53k and the average of the right and left WCA was considered.

Most of the mechanical tests were conducted more than once under the same conditions, and the average results were taken. The Origin Lab Programme, Version 9.4, was utilized for the execution and analysis of each plot. The WCA values and all examined mechanical parameter errors did not surpass 5%.

## 3. Results and Discussion

### 3.1. AFM Analysis

**Figure 3:**
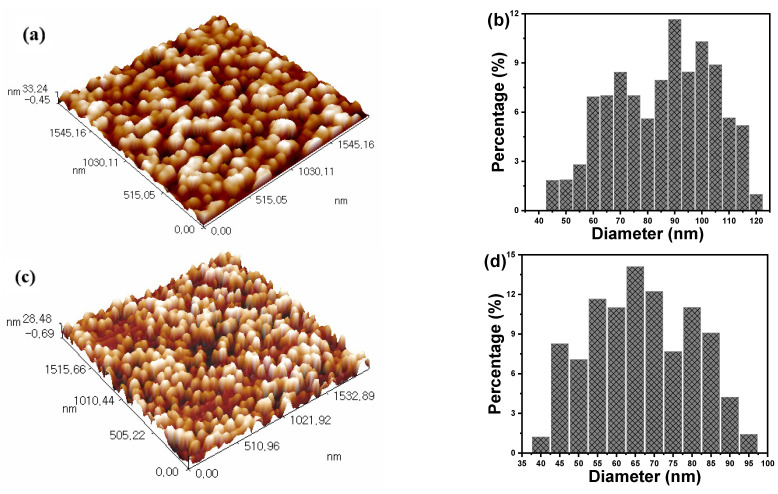
Three-dimensional AFM topographic images and granularity normal distribution charts of (**a**,**b**) silica and (**c**,**d**) cement nanoparticles.

AFM analysis is a powerful approach for giving integrated information about the tested materials’ surface shape, particle size, and particle distribution. Figure 3 illustrates the 3D AFM images and particle distributions of silica and cement powder. The brightest areas in the AFM images have the most nodules, which are generally located in the center of the image. This observation may indicate the probability of nanoparticle aggregation as well as denser morphology in various areas. The size of the particles, on the other hand, was equally distributed throughout the image, indicating a homogeneous dispersion of silica and cement nanoparticles. Silica and cement have average particle sizes of 82 ± 20 nm and 68 ± 15 nm, respectively. The roughness of the nanoparticles studied was calculated to be 7.21 nm for silica nanoparticles and 6.95 nm for cement nanoparticles.

### 3.2. Abrasion Wear Resistance

**Figure 4:**
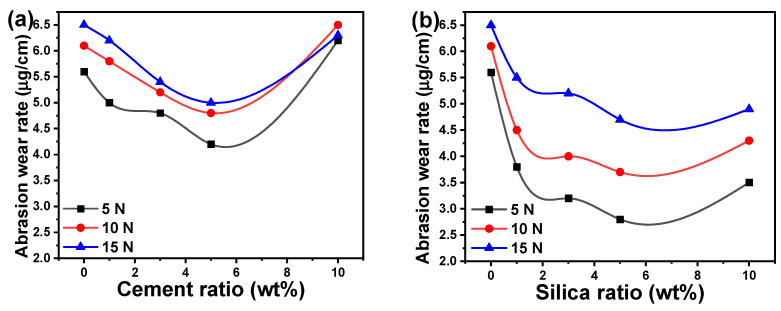
Abrasion wear rate of acrylic composites versus the weight ratio of (**a**) cement and (**b**) silica nanoparticles.

The value of abrasion wear resistance for any polymer is determined by the wear mechanisms, the abrasive test technique, and the bulk and surface characteristics of the investigated samples. The abrasion test was used in this work to determine the abrasion resistance of acrylic polymer-based composites. The influence of the abrasion wear rate of acrylic polymer composites on cement ratio and applied force is depicted in Figure 4a. As the cement concentration was raised to 5 wt%, the abrasion wear rate of acrylic/cement composites was significantly decreased; however, at a ratio of 10 wt%, the rate of wear was significantly increased. For instance, for a constant applied force of 5 N, the abrasion wear rate of acrylic polymer composites decreased from 5.6 μg/cm for the pure acrylic polymer to 4.2 μg/cm for a loading cement ratio of 5 wt% and then increased to 6.2 μg/cm for a loading cement ratio of 10 wt%. Conversely, for all composites with a cement ratio between 0 and 5 wt%, the abrasion wear rate of acrylic polymer composites increased steadily as the applied force increased. Interestingly, the acrylic polymer composites with a 10 wt% cement ratio were unaffected by the applied force and the shift in the experimental error range. The abrasion wear rate of acrylic/cement composites consisting of 10 wt% cement equals 6.2, 6.5, and 6.3 μg/cm for applied stresses of 5, 10, and 15 N, respectively.

The influence of the abrasion wear rate of acrylic polymer composites on silica ratio and applied force is shown in Figure 4b. The abrasion wear rate of acrylic polymer composites was greatly reduced by the increase in the silica content to 5 wt% and subsequently significantly increased at a concentration of 10 wt%, showing a similar trend to acrylic/cement composites. At a constant applied force of 5 N, for example, the abrasion wear rate of acrylic polymer composites was reduced from 5.6 μg/cm for pure acrylic polymer to 2.8 μg/cm for a loading silica ratio of 5 wt%, and subsequently raised to 3.5 μg/cm for a loading silica ratio of 10 wt%. The abrasion wear rate of acrylic composites, on the other hand, rose constantly as the applied force increased for all composites with a silica ratio in all studied ranges. For applied forces of 5, 10, and 15 N, the abrasion wear rate of acrylic composites composed of 10 wt% silica equaled 3.5, 4.3, and 4.9 μg/cm, respectively. The abrasion wear rate values recorded for acrylic/silica composites were lower than those recorded for acrylic/cement composites. The observed behavior could be explained by silica’s greater particle size compared to cement nanoparticles, as well as the quality of filler dispersion inside the acrylic polymer matrix. In addition, this finding may be likened to the incorporation of silica or cement into the polymeric matrix. In other words, doping with nanofiller alters the morphological characteristics of the coverage due to the enhanced interaction of silica or cement with the coating configuration, resulting in a coating that appears to be more compact and less abraded than the coating without silica and cement. The coating’s compactness grows as the ratio of silica and cement increases. This indicates that the interface surface contact between silica or cement and the polymer coating matrix offers abrasion resistance and effective dispersion of the nanofiller inside the polymer coating. The wear rate of the acrylic polymer increases with each additional load applied in all samples. Furthermore, the abrasion wear rate of acrylic polymer composites and epoxy/ZrO_2_ composites with varied filler ratios demonstrated distinct behaviors [17]. The wear rate decreases with increasing SiO_2_ particle size because when SiO_2_ particles are small enough, more SiO_2_ nanoparticles can be dispersed on the friction layer, which is consistent with other studies [49]. Other research studies have found that incorporating nanofiller inside acrylic polymer matrices such as silica or cement nanoparticles reduces wear rate [50,51,52].

### 3.3. Impact Strength and Hardness

**Figure 5:**
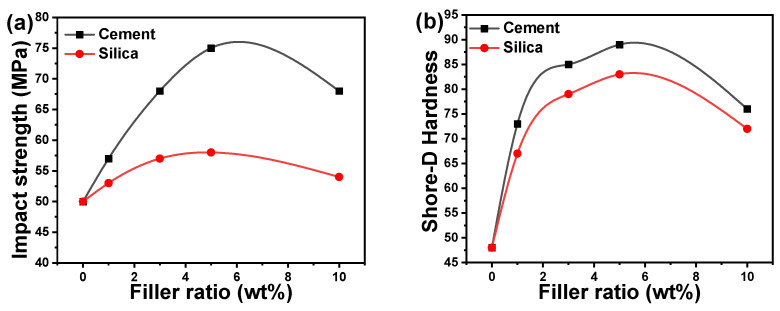
(**a**) Impact strength and (**b**) Shore-D hardness for acrylic composites as a function of filler ratios.

It is generally known that the functionalization of materials to be geometrically complicated is usually demonstrated for greater density and the highest impact strength of the substance. Doping polymers with fillers such as cement or silica, for example, can be regarded as a beneficial condition since it minimizes the free stretching of long chains of the polymer, and hence decreases the adaptability of the organic network and enhances its mechanical and thermal characteristics [53]. Figure 5a depicts the impact strength of acrylic/cement and acrylic/silica composites versus the cement and silica ratios. The impact strength increased with the increase in the cement or silica ratios up to 5 wt% and subsequently decreased with additional increases in the filler ratios up to 10 wt%. The acrylic/cement composites had higher impact strengths than acrylic/silica composites: the impact strength was 1.5 times higher for acrylic/cement composites than for acrylic/silica composites. The higher increase in mechanical characteristics such as impact strength by cement loading into acrylic polymer composites may be ascribed to the smaller size of cement nanoparticles compared with silica nanoparticles, resulting in a more uniform distribution of cement nanoparticles and decreased aggregation in the polymer matrix. In addition, the better mechanical properties of acrylic composites could be attributed to the good interaction of the acrylic coating with cement and silica nanoparticles. In other words, improved covalent bonding between the nanofillers and the polymer matrix might explain the considerable increase in impact strength and other mechanical characteristics. Various kinds of covalent flexible networks are found and categorized into two groups (dissociation and association) according to the mechanism by which their reactions work [41]. The dissociation type, in which the bond first breaks and then re-forms after a short period, results in a rapid fall in the polymer network’s crosslinking density following the reaction. This can potentially lead to the depolymerization of the polymer network into a linear chain, which could significantly alter the mechanical strength [44]. On the other hand, bond breaking and reformation happen concurrently in the association type, and the crosslinking density stays largely the same. Nevertheless, at higher cement and silica ratios (>5 wt%), the fillers could be aggregated or agglomerated inside the polymer matrix due to inadequate filler dispersion, hence resulting in brittle behavior and decreasing the impact strength [54]. The aggregation of acrylic polymer composites containing 10 wt% cement or silica resulted in a decrease in impact strength owing to the larger surface area of the accumulated nanofillers and the stress concentration around the aggregation, which might lead to a tendency to crack.

The computed Shore-D hardness values of acrylic composites are displayed in Figure 5b. Impact strength and the Shore-D hardness trend with filler ratio exhibit comparable behavior. The doping of acrylic polymer with silica and cement nanoparticles increased its hardness roughly two-fold at a loading ratio of 2 wt%. Then, the improvement rate in hardness became smaller as the doping content was raised to 3 and 5 wt%. The highest enhancement was seen for the optimal filling ratio of 5 wt% while increasing the doping ratio to 10 wt% resulted in a drop in the Shore-D hardness value. The doping of acrylic polymer with cement or silica nanoparticle filler resulted in an increased hardness for all investigated acrylic composites in the 0–5 wt% ratio range. The most uniform and homogenous dispersion of silica and cement nanoparticles, as well as strong interfacial contact between cement and silica and acrylic polymer matrix, was obtained at 5 wt% ratios, which corresponds to higher Shore-D hardness. The improved mechanical properties of acrylic polymer composites were affected by additional filler additives, which resulted in greater surface area and hence lower scratch resistance [19]. The Shore-D hardness of the acrylic polymer improved from 48 to 89 and 83 for the acrylic polymer composites incorporating 5 wt% cement or silica, respectively. The Shore-D hardness value decreases when the cement or silica content increases over 5 wt%. The initially enhanced Shore-D hardness shows less ductility and becomes brittle when additional cement and silica are loaded. Doping acrylic polymers with cement nanoparticles improves Shore-D hardness more than doping with silica because cement particles are smaller, resulting in better cement dispersion in the acrylic polymer matrix.

The addition of silica nanoparticles into a polymer matrix frequently increases the mechanical parameters of polymers such as acrylic polymer in this study and polyimide matrix composites in another investigation [49]. The incorporation of silica nanoparticles into the acrylic polymer increased mechanical characteristics, which is consistent with earlier research [55,56]. The results of previous studies support the impact of nanosized silica filing on mechanical polymer composites [57]. In general, the incorporation of silica nanoparticles into polymers has a considerable impact on the specimen’s composite material’s wear behavior tests, impact strength, fracture toughness, and shore-D hardness. The hand-lay-up approach was utilized to make an unsaturated polyester resin matrix reinforced with 1, 3, and 5 wt% silica nanoparticles, which had a particle size of ~45 nm [57]. The results of the mentioned study demonstrated similar behavior to our investigation, in that the composite containing 5 wt% SiO_2_ improved the above-mentioned mechanical parameters. The addition of cement particles improved the mechanical characteristics of the acrylic polymer more than silica nanoparticles due to the smaller particle sizes.

### 3.4. Dielectric Strength and Thermal Insulation

**Figure 6:**
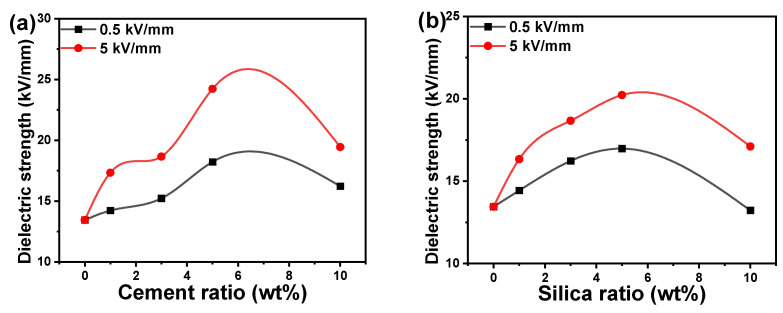
Dielectric strength of acrylic polymer composites versus the weight ratio of (**a**) cement and (**b**) silica nanoparticles.

The dielectric strength of polymer composites is usually determined by the type and quantity of incorporated filler nanoparticles. Figure 6a,b illustrate the dielectric strengths of acrylic polymer composites including cement or silica nanofiller at two different applied electric fields of 0.5 and 5 kV/mm. The dielectric strength of acrylic polymer composites was enhanced by raising the filler ratio to 5 wt%, then lowered at a filling ratio of 10 wt%. The drop in dielectric strength at a ratio of 10 wt% revealed that the filler nanoparticles form agglomerations rapidly, even at modest loadings, and act like micro-sized particles, reducing the dielectric strength. Similar to other parameters examined, doping with cement nanoparticles yields higher dielectric strength than acrylic polymer composites containing silica. Filler agglomerations can be reduced by covering the surface of the nanoparticles with an appropriate coating to make them more friendly with the polymer matrix. However, in this study, an uncoated and polar surface of the cement and silica nanoparticles was preferred. The composite’s matrix phase of the polymer should be considered the whole of its three components, each of which contributes individually to the arrangement of the matrix structure [48]. The boundary layer, which is in direct contact with the filler surface, is one of the constituent parts. Furthermore, between the polymer layer and the boundary layer, there may be a transition layer with less tightly packed polymer chains [54]. The final composite’s characteristics can be influenced by structural and other properties of these layers, which will vary greatly from the polymer properties in volume. The acrylic polymer containing 5 wt% cement and silica nanoparticles demonstrated the highest dielectric strength and therefore could serve as a polymer matrix for voltage stabilization.

The heat released from the dielectric material when an electric field is applied to the dielectric is ascribed to the loss of insulation caused by leakage currents; then, the impact of fusion, permeability, and fractures appears on the dielectric and is determined by the frequency of the voltage and the duration of the reaction. The dielectric strength was measured at the lowest electric field of 0.5 kV/mm and the highest electric field of 5 kV/mm and was increased by increasing the electric field. Similar to most of the mechanical properties, the dielectric strength of acrylic/cement composites is greater than that of the acrylic/silica composites, which could be ascribed to the smaller nanoparticle size of cement filler compared to silica nanoparticles.

The distribution of pore sizes, the total porosity of the material, and the density of ions in the pore solution all have a significant impact on cement-based systems’ electric impedance [18]. The ionic concentration at a particular water-to-cement ratio in cement paste influences the electric impedance response during the initial hydration phase. This is because low impedance is produced when high porosity and high ionic concentration are combined. Further, it is anticipated that the impedance will rise as the porosity and pore size decrease [35]. As the ratio increased, the dielectric constant fell for cement pastes with varying ratios of water to cement, suggesting a relationship between the dielectric constant and the variation in the solid phase fraction by volume. Recently, there has been an increased interest in identifying the electric impedance and dielectric constant of low-porosity cement [16,18,47].

In this study, the dielectric strengths of acrylic polymer composites were higher than those of other polymer systems such as polyester resin doped with 0–15 wt% MgO nanoparticles [47]. The increase in dielectric strength caused by the inclusion of silica or cement nanoparticles in acrylic polymers might be due to their differences in conductivity, which increased the possibility of micro-capacitor generation, resulting in higher dielectric strength [58]. The increase in filler ratio to 10 wt% resulted in a drop in dielectric strength due to filler particle aggregation, and the number of micro-capacitors was reduced. The observation of dielectric strength change for an acrylic polymer with nanoparticle addition is consistent with prior findings [58].

Pure polymer materials have weak thermal and electrical conductivities. The electrical and thermal conductivity of these materials can be enhanced by incorporating conductive fillers such as CNT, MWCNT, graphene, etc., or metals such as Ag, Cu, etc. Integration of a polymer with an insulator filler is appropriate when it is important to reduce the thermal and electrical conductivities of the polymer. Even though polymers are categorized as either good thermal insulators or low thermal conductivity materials (0.1–0.5 W.m.K^−1^), their single chains exhibit thermal conductivities that are substantially higher than their bulk values. Polymers have lower degrees of thermal conductivity due to their lower degrees of crystallinity [59].

**Figure 7:**
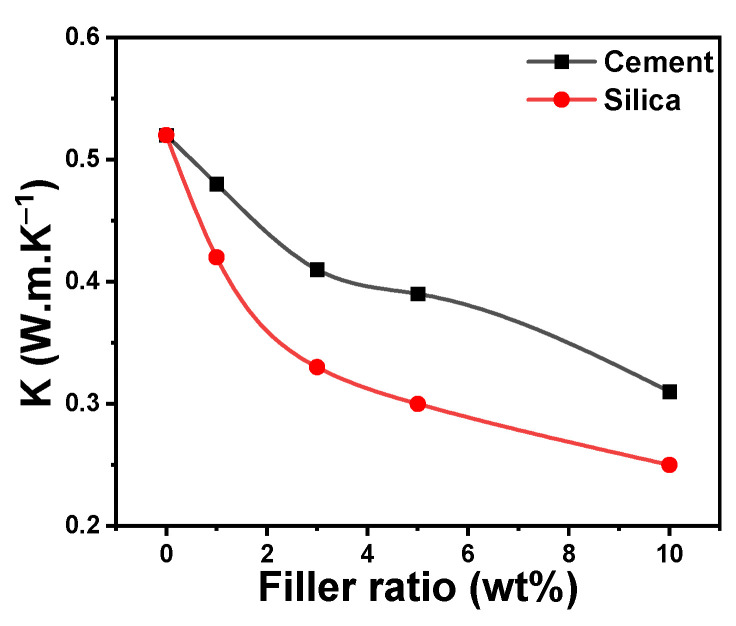
Thermal insulation, or thermal conductivity (K), of acrylic composites versus the filler ratio.

Figure 7 illustrates the thermal insulation or thermal conductivity (K) of acrylic polymer composites. The value of K increased with the increase in the filler nanoparticle concentration due to the thermal insulation properties of the incorporated ceramic material. The thermal conductivity coefficient for the acrylic composites decreased as the cement or silica ratios increased. The anticipated values for K are higher for cement-containing acrylic polymer composites compared to acrylic/silica composites. In other words, the thermal insulation of acrylic/cement composites is greater than that of acrylic/silica composites. Concrete usage can benefit from the thermal insulation provided by acrylic polymer doped with cement nanoparticles. For instance, it demonstrated the capacity to transport heat gains or losses through the building envelope when exposed to hard circumstances, low thermal conductivity, correspondingly reduced thermal diffusivity, and high specific heat. These characteristics result from variations in the insulating materials employed, such as concrete’s density, temperature, and moisture content [60].

### 3.5. Surface Roughness and Water Contact Angle

**Figure 8:**
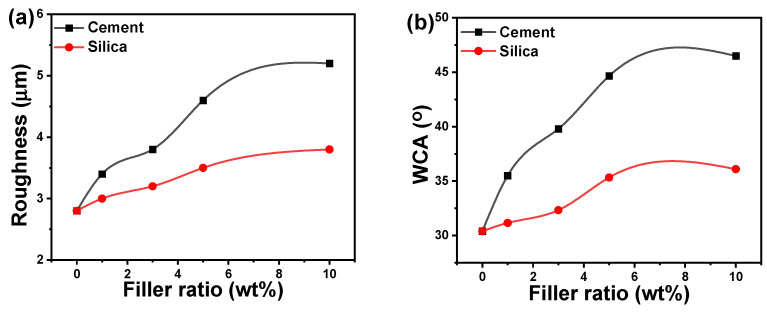
(**a**) Surface roughness and (**b**) water contact angle (WCA) of acrylic composites as a function of filler ratio.

The arithmetic average height or roughness (*R_a_*) values for acrylic polymer composites as a function of cement and silicon ratios are illustrated in Figure 8a to investigate the impact of cement and silica concentration on surface roughness. The plot shows that increasing the ratios of silica and cement fillers causes a rise in roughness, revealing that the acrylic polymer surface roughness values rose considerably. The highest determined roughness values for an acrylic polymer containing 10 wt% silica and cement are 3.5 and 5.4 μm, respectively. The highest surface roughness value was obtained at the maximum filling ratio (10 wt%) could be ascribed to height distribution differences between the polymer and the nanoparticles. Furthermore, the surface roughness of acrylic/cement composites is higher than that of acrylic/silica composites, which could be attributable to the smaller size of cement nanoparticles compared to silica nanoparticles. It is possible to conclude that the addition of polymer with higher roughness filler results in a lower roughness polymer composite surface based on the individual cement and silica’s roughness as well as the roughness of the composites of acrylic/cement and acrylic/silica.

The WCA of acrylic/cement and acrylic/silica compositions as a function of cement and silica ratios is shown in Figure 8b. In general, the surface roughness and WCA behaviors of acrylic composites exhibit identical behaviors with the filler ratios. The addition of cement and silica nanoparticles to the acrylic polymer matrix increased the surface roughness and the WCA. The typical WCA value for pure acrylic polymers is 30.4 ± 0.3°, emphasizing their hydrophilic behavior. By increasing the silica or cement filler in the acrylic polymer matrix, the WCA angle was dramatically increased. At a filler ratio of 5 wt%, the difference in WCA becomes insignificant. In the 5–10 wt% ratio range, the maximum WCA recorded is 45.6 ± 0.9° for acrylic/cement composites and 35.7 ± 0.4° for acrylic/silica composites. The addition of silica and cement enhances the WCA of pure acrylic polymers, consistent with previous research [18,51].

According to the analysis above, incorporating cement and silica nanoparticles into the polymer matrix significantly enhanced their mechanical characteristics as well as their dielectric strength and thermal insulation. Unfortunately, further work is still needed to enhance the WCA and wettability of the investigated acrylic polymer composites before they can be applied for waterproofing purposes. Higher WCA is also needed for other technical uses. As a result, we provide a great strategy—plasma treatment—in the following section for modifying the surface and enhancing the hydrophobic characteristics of acrylic composite surfaces.

The distinction in characteristics between acrylic/cement and acrylic/silica composites is not related to nanosize only, but also to the specifications and properties of this material, which provide high specifications for the composite. In the majority of studied properties, the acrylic/cement composite outperformed the acrylic/silica composite. All experiments demonstrated an improvement as the cement and silica proportions increased. The optimal proportion was 5 wt%. When the percentage increased to 10 wt%, the characteristics dropped due to cement or silica nanoparticle agglomeration. Also, the abrasion wear rate rose as the load increased. We found that when the load was 5 N, the abrasion wear rate was lower than when the load was 10 N or 15 N. However, it exhibited perfect characteristics when the load was 5 N. This is only decided by the application, and it is well understood that increasing the amount of load increases the rate of abrasion wear.

In general, acrylic resins have weak mechanical and physical characteristics, limiting their applicability in other applications. As a result, the study of the features of reinforced acrylic doped with various fillers to make acrylic composites has been a popular research issue for this material, including aspects such as improving composite mechanical properties. Various fillers, including glass beads, SiO_2_, and carbon fibers, could be utilized to improve the mechanical characteristics of polymer composites [50,52]. Furthermore, the hardening time and mechanical properties of polymer composites are mainly affected by the type of filler, its ratio, curing temperature, and aggregate quantity [15]. For example, the curing temperature is an important aspect affecting hardening and strength development properties. Large amounts of polymers may generate a special spatial network structure that encases the cement-based elements. In a composite material system, a small amount of polymer cannot entirely enclose the cement-based material and act as its own spatial network [18]. Following this, the 3D interpenetrating network structure can be generated by the polymer matrix and the cement hydration products [28]. The polymer will get distributed in the hardened cement as the amount of polymer decreases and is unable to form a continuous film structure. In addition to the standard film theory, there are other mechanisms involving chemical bonding between polymers and cement or cement hydration products in the modification mechanism of polymers on cement-based composites [16]. On the other hand, most people agree that the physical influence on the characteristics of cement-based compounds modified with polymers outweighs the chemical effects [32].

### 3.6. Characterization of Plasma Jet and Its Usage for Surface Modification

In this section, we investigate the optical emission spectrum characteristics of the plasma jet and investigate its dependence on the Ar flow rate. Secondly, the plasma jet, operated under different conditions of Ar flow rate and exposure time, was employed to modify the surface of acrylic composites and enhance their roughness and hydrophobic properties.

**Figure 9:**
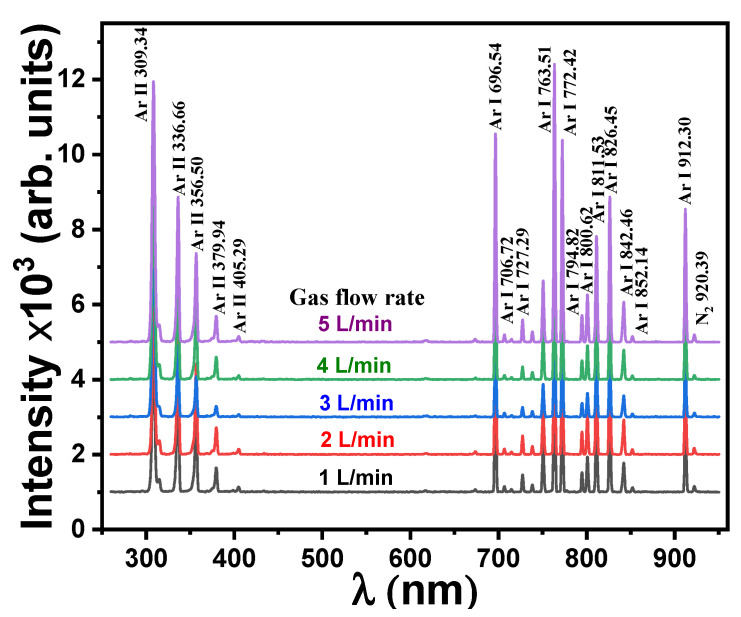
Optical emission spectra for different gas flow rates.

Spectroscopy is a practical tool for calculating the temperature and intensity of the Ar plasma jet in the wavelength range of 250–950 nm. Figure 9 illustrates the intensity and distribution of the plasma emission spectra obtained for different gas flow rates. The spectra revealed many peaks ascribed to Ar I and Ar II, which is consistent with National Institute of Standards and Technology (NIST) data [41]. Several peaks ascribed to Ar I are detected at wavelengths of 696.54, 706.72, 727.29, 763.51, 772.42, 794.82, 800.67, 811.53, 826.45, 842.46, and 852.14 nm for different gas flow rates. Also, there are a few peaks assigned to Ar II that are centered at wavelengths equal to 309.34, 336.66, 356.50, 379.94, and 405.29 nm. Moreover, a single peak belongs to N I observed at 920.39 nm due to the plasma being generated in the atmosphere.

**Figure 10:**
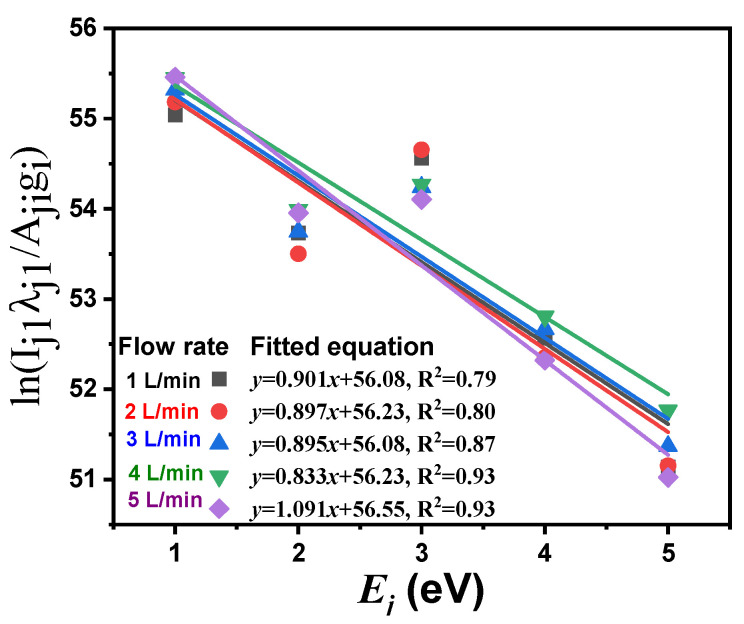
Boltzmann plot ($Ln(\frac{I_{ji}\;\lambda_{ji}}{A_{ji}\;g_{i}}$) versus $(E_{j}$) for different Ar flow rates.

Figure 10 represents the $Ln(\frac{I_{ji}\;\lambda_{ji}}{A_{ji}\;g_{i}}$) as a function of ($E_{j}$). The density of electrons ($n_{e}$) was calculated via stark broadening using the following equation [42]: $n_{e}=\left(\frac{\Delta \lambda }{2\omega_{s}}\right)N_{r}$, where Δ*λ* is the full width at half maximum of the line, $\omega_{s}$ is the Stark broadening parameter, and $N_{r}$ is the reference electron density. The statistical coefficient (R^2^) indicates the quality of fitted lines and equals a value between 0.8 and 0.93 in this study.

In general, to improve the wettability of acrylic/silica and acrylic/cement composites, the flow rate of Ar gas utilized to generate the plasma torch and the exposure time were optimized in this study. The Boltzmann plot (Figure 10) and the Stark broadening approach were used for calculating the electron temperature ($T_{e}$) and electron density ($n_{e}$). The $T_{e}$ is the most important parameter that distinguishes plasmas. It is a sort of energy that is important in the plasma because $T_{e}$ determines plasma ionization. Furthermore, because plasma characteristics are dependent on $n_{e}$ in the plasma medium, its diagnostics take precedence. The electrons play the main role in the processes within plasmas, so the difference in $n_{e}\;$ produces different plasmas and thus different applications. To describe the plasma, the Debye length ($\lambda_{D}$) and plasma frequency ($\omega_{P}$) could be estimated as follows [10,61]: $\lambda_{D}=\sqrt{\frac{k_{B}T_{e}}{4\pi e^{2}n_{e}}}$ and $\omega_{P}=\sqrt{\frac{n_{e}e^{2}}{\varepsilon_{0}m_{e}}}$, where $k_{B}$ is the Boltzmann constant, $m_{e}$ is the mass of the electron, and $\varepsilon_{0}$ is the electric permittivity for free space.

**Figure 11:**
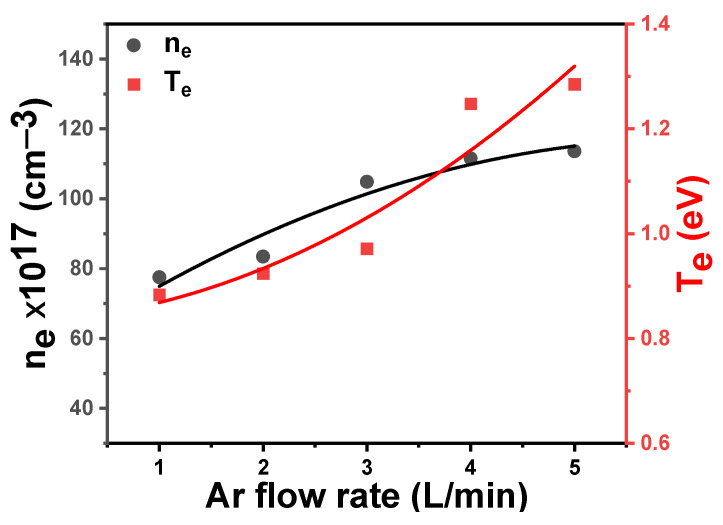
Variation of electron density ($n_{e}$) and electron temperature ($T_{e}$) versus the Ar flow rate.

The behavior of the jetting plasma coefficients, especially $T_{e}\;$ and $n_{e}$, at different Ar flow rates has been determined. The effects of Ar flow rate on plasma characteristics are summarized in Table 1. As is evident from Figure 11 and Table 1, $T_{e}$ and $n_{e}$ increase with the increase in the flow rate of Ar. Increasing the flow rate of Ar gas to the plasma region means an increase in the number of atoms pushed to the applied high electric field, which leads to acceleration of electrons and them gaining high energy, which is enough to cause ionizing collisions of gas atoms, thus the electron density increases. Exposing the electrons to a high potential difference supplies them with energy, and hence $T_{e}$ rises. The energy of these electrons and other plasma components helps to motivate the acrylic composite surface to improve its hydrophobic properties by changing the chemical properties and roughness of its surface layer. The influence of the plasma treatment on the surface of the acrylic composites can be improved by increasing the flow rate of the Ar as well as by increasing the exposure time of the surface of the sample to the plasma. The plasma increases the hydrophobicity of the sample without causing any damage to the surface of the sample or leaving toxic residues on it.

From Table 1, it can be observed that an increase in Debye length ($\lambda_{D}$), the Debye number (*N_D_*), and the plasma frequency ($\omega_{p}$) can be interpreted as an increase according to the observed increase in $T_{e}$ and $n_{e}$ with the increase in the Ar flow rate in the plasma jet system. The Debye length is inversely proportional to the square root of $n_{e}$ and directly proportional to $T_{e}$. An increase in $n_{e}$ and $T_{e}$ because of an increase in the Ar flow rate means that $\lambda_{D}\;$ also increases. The higher electron density means a larger screening effect and higher electron temperature contributes to greater thermal electron movement, which leads to expansion of the area of effect around the charged particle.

**Figure 12:**
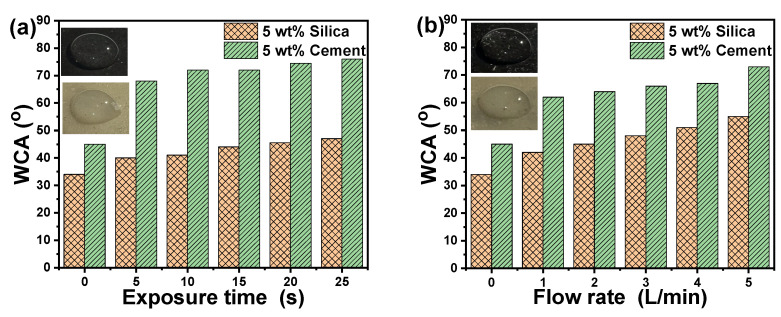
Effect of (**a**) exposure time and (**b**) Ar flow rate of plasma on the WCA of acrylic polymer composites. The inserted images show the WCA measurement at an exposure time of 15 s or an Ar flow rate of 3 L/min as examples.

The WCA for acrylic composites containing 5 wt% silica and 5 wt% cement is 35.3° and 44.7°, respectively, and is used as a reference sample before plasma treatment for various exposure durations and Ar flow rates. Figure 12 depicts the effect of plasma treatment on the WCA and the hydrophobic properties of the produced acrylic composites. For example, we chose two distinct materials to investigate the influence of plasma treatment on their wettability: acrylic polymer consisting of 5 wt% silica (beige samples) and 5 wt% cement (black samples). Figure 12a depicts the effect of plasma treatment exposure duration ranging from 5 to 25 s at a fixed Ar flow rate of 5 L/min. Figure 12b depicts the effect of plasma treatment with an Ar gas flow rate ranging from 1 to 5 L/min for the aforementioned acrylic composites at a constant plasma exposure time of 25 s. The value of the WCA is commonly seen to alter by 1.3 and 1.6 times as the period of plasma exposure or the Ar flow rate is changed for acrylic composites containing 5 wt% silica or cement, respectively. Also, under the same treatment circumstances, the presence of cement exhibited larger WCA than acrylic/silica composites. The exposure duration and Ar flow rate had a considerable impact on the WCA of the tested acrylic polymer composites.

The WCA results show that plasma activation, independent of additives, was successful in making the surface of the acrylic polymer composites hydrophobic by increasing its surface energy [10,62]. Plasma treatment may be utilized for surface treatment and activation and has the same impact on wettability as it does on other materials. The effects of plasma treatment on hydrophobic properties may be caused by a change in the surface composition, as Ar plasma may react with the surface of polymer composites, replacing oxygen with Ar [63]. Another possible explanation for the change in WCA caused by plasma treatment is the alteration in surface morphology and roughness. Plasma treatment of acrylic polymer composites successfully increased surface roughness, resulting in improved hydrophobic properties, which is consistent with previous research on copper-cleaning surfaces [64]. Increasing the plasma exposure period reduces the wettability of the acrylic polymer composite in this study, consistent with previous research on surgical gowns [37].

**Figure 13:**
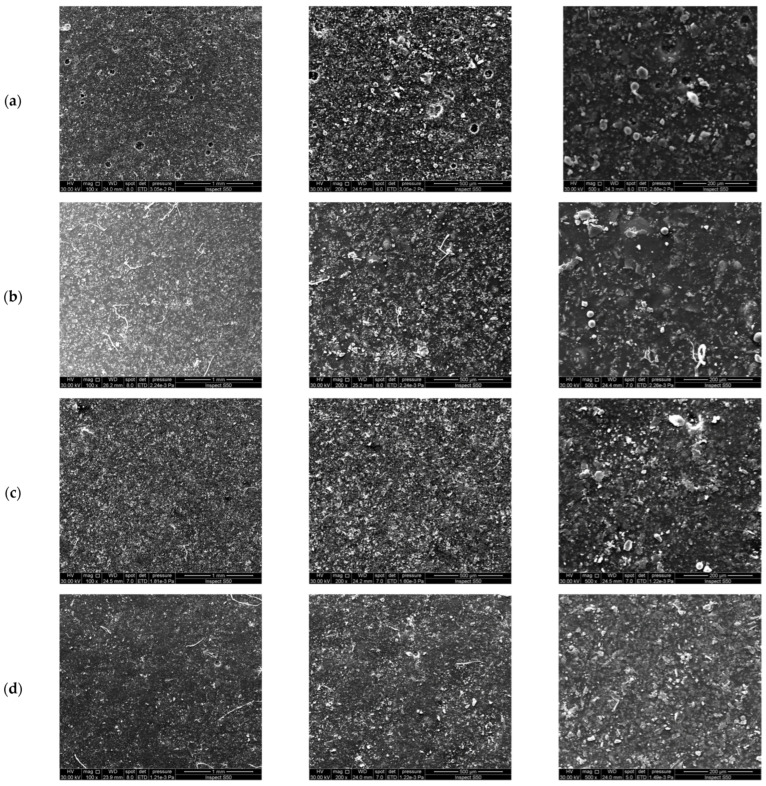
SEM images of acrylic composites incorporated with 5 wt% cement (**a**) before and (**b**) after plasma treatment observed under different magnifications. SEM images of acrylic composites incorporated with 5 wt% silica (**c**) before and (**d**) after plasma treatment. Plasma treatment was performed at an exposure time of 25 s and an Ar gas flow rate of 5 L/min.

The aggregation of cement and silica nanofiller in an acrylic composite coating matrix was evaluated using the SEM technique. SEM images of acrylic composites doped with 5 wt% cement or silica before and after plasma treatment are shown in Figure 13. The mentioned composites are chosen because they exhibit the best mechanical characteristics and higher WCA. The plasma treatment of acrylic polymer composites doped with 5 wt% cement or silica is conducted at a constant exposure duration of 25 s and an Ar flow rate of 5 L/min. Figure 13a,b as well as Figure 13c,d show SEM images of acrylic/cement and acrylic/silica composites before and after plasma treatment, respectively. The effect of plasma treatment can be seen clearly on the surface of the samples by comparing the SEM images of the same sample before and after plasma treatment. The effect of plasma on surfaces and its ability to make them hydrophobic was confirmed by measured WCA. It is well known that plasma is an ionized gas composed of ions, electrons, and neutral particles [39,65]. The exposure of the surface of acrylic composites to plasma radiation results in various physical and chemical changes that can alter its properties, i.e., wettability [34]. In addition, plasma treatment activates the surface by bombarding it with energetic ions and electrons, which leads to the breaking of chemical bonds and the removal of contaminants or organic substances present on the surface [64]. The energetic particles in the plasma remove any adsorbed or loosely bound molecules from the surface, resulting in a clean and chemically reactive surface. The plasma jet caused a remarkable modification of the surface of the composites. Reactive species such as free radicals, ions, and excited molecules are generated upon plasma treatment. These species can interact with the surface, leading to chemical modifications. Thus, the plasma can generate functional groups or change the chemical composition of the surface. Accordingly, plasma treatment can effectively modify acrylic composite surfaces to make them hydrophobic. The specific parameters for surface treatment by plasma, such as Ar flow rate, treatment time, and substrate material, are important factors in controlling the desired surface’s properties. The influence of surface treatment by plasma for the acrylic/cement composites was greater than for the acrylic/silica composites. The observation emphasized by the SEM test is consistent with the WCA measurements.

The cement, as well as silica nanoparticles, exhibit spherical-like shapes inside the acyclic polymer matrix, whereas their sizes vary as a result of varied amounts of agglomeration of various particles inside the acrylic polymer. The cement and silica microstructures inside the polymer matrices are consistent with previously published research [18]. Even though the dispersed filler is formed of varied particle sizes inside the polymer matrix, it displays a homogeneous distribution of cement and silica filler within the acrylic polymer matrix. Furthermore, examination using SEM revealed an improvement in the binding strength between the polymer and the surrounding matrix. One of the most important aspects impacting mechanical properties and concrete durability is the high and homogeneous distribution of the filler inside the acrylic polymer matrix. Plasma treatment affects surface morphology, roughness, and smoothness. The measured WCA increased as the surface was exposed to plasma treatment and became less rough and smoother after plasma treatment. The particle size of acrylic polymer doped with cement varied from 2 to 5 μm before plasma treatment, and its range changed to 0.6 to 2 μm after plasma treatment. The silica-doped acrylic polymer’s particle size before plasma treatment ranged between 0.7 and 2.5 μm and between 0.1 and 2 μm after the plasma treatment.

Plasma treatment in this context refers to non-thermal plasma, specifically the dielectric barrier discharge plasma jet. It is widely employed for surface modification due to its ability to enhance surface properties without significantly altering the material’s bulk characteristics [44]. However, its impact on mechanical properties like tensile strength, hardness, and impact resistance is minimal. This is because plasma operates on the material’s surface, causing only surface-level reactions, such as cross-linking and the interaction of functional groups, without affecting the internal structure of the polymer or composite. The observed increase in mechanical characteristics after plasma treatment appears to be due to better adhesive bonding quality and adhesion properties [41]. In addition to bulk characteristics, surface attributes like hydrophobicity, surface tension, and roughness are critical to the effective performance of any item made from that material [33]. Plasma has the following effects: surface washing, elimination of organic pollutants, surface deterioration (etching), cross-linking of polymer chains, and alteration of the surface’s functional groups [34,35].

If the sizes of reinforcement materials are micro or macro-scale, the benefits of employing them as fillers are minimal [28]. Furthermore, due to the filler material’s micro size, it has a higher density, resulting in heavier composites, limiting its use in a number of applications [38]. It is worth noting that the use of filler material in composite construction was originally employed to reduce the cost of polymeric components [19].

## 4. Conclusions

Pure acrylic and acrylic composites were made by the casting method, and the influence of incorporation type and concentration on mechanical and thermoelectric insulation qualities was investigated. The surface was treated using plasma to improve its hydrophobic properties. The maximum impact strength, Shore-D hardness, dielectric strength, and roughness of acrylic polymer were achieved for 5 wt% of cement or silica nanoparticles ratio. The mechanical characteristics of the acrylic composites decreased with the incorporation of 10 wt% of cement and silica nanofiller. Aggregation of the nanomaterial (silica or cement), which diminished the characteristics of the composite and produced subpar outcomes, was the cause of the 10% ratio’s failure. Acrylic/cement composites demonstrated superior mechanical, thermal, and hydrophobic characteristics compared to acrylic/silica composites owing to smaller cement filler sizes and thermoelectric properties. The impact of plasma treatment on the surface hydrophobic properties of acrylic composites containing 5 wt% silica or cement was investigated. The examined acrylic polymer composites show great improvement in hydrophobic properties, where the WCA increased two-fold over the WCA of pure acrylic polymer. The influence of plasma exposure duration and Ar flow rate on the WCA value was explored and found to be significant. The developed acrylic polymer with cement and silica nanofillers has potential applications in construction, the environment, and architecture.

## Figures and Tables

**Table 1:** Plasma parameters for different flow rates of Ar gas.

Flow Rate (L/min)	$T_{e}$ (eV)	$n_{e}$ × 10^16^ (cm^−1^)	$N_{D}$	$\omega_{p}$ × 10^11^ (rad/s)	$\lambda_{D}$ × 10^−7^ (cm)
1	0.892	77.99	1645	499.15	7.95
2	0.927	83.63	1683	516.86	7.82
3	0.976	105.11	1622	579.45	7.16
4	1.253	111.97	2285	598.08	7.86
5	1.292	112.85	2383	600.42	7.95

## Data Availability

The original contributions presented in the study are included in the article, further inquiries can be directed to the corresponding author.
